# Wearable energy-dense and power-dense supercapacitor yarns enabled by scalable graphene–metallic textile composite electrodes

**DOI:** 10.1038/ncomms8260

**Published:** 2015-06-11

**Authors:** Libin Liu, You Yu, Casey Yan, Kan Li, Zijian Zheng

**Affiliations:** 1 Nanotechnology Center, Institute of Textiles and Clothing, The Hong Kong Polytechnic University, Hung Hom, Kowloon, Hong Kong, China; 2 Shandong Provincial Key Laboratory of Fine Chemicals, Key Laboratory of Fine Chemicals in Universities of Shandong, Qilu University of Technology, Jinan 250353, China; 3 Advanced Research Centre for Fashion and Textiles, The Hong Kong Polytechnic University Shenzhen Research Institute, Shenzhen 518000, China

## Abstract

One-dimensional flexible supercapacitor yarns are of considerable interest for future wearable electronics. The bottleneck in this field is how to develop devices of high energy and power density, by using economically viable materials and scalable fabrication technologies. Here we report a hierarchical graphene–metallic textile composite electrode concept to address this challenge. The hierarchical composite electrodes consist of low-cost graphene sheets immobilized on the surface of Ni-coated cotton yarns, which are fabricated by highly scalable electroless deposition of Ni and electrochemical deposition of graphene on commercial cotton yarns. Remarkably, the volumetric energy density and power density of the all solid-state supercapacitor yarn made of one pair of these composite electrodes are 6.1 mWh cm^−3^ and 1,400 mW cm^−3^, respectively. In addition, this SC yarn is lightweight, highly flexible, strong, durable in life cycle and bending fatigue tests, and integratable into various wearable electronic devices.

## Abstract (web summary)

It is challenging to fabricate flexible and wearable supercapacitors with high energy and power densities using cost-effective materials and scalable processes. Here, the authors present a supercapacitor yarn based on graphene and metallic textile composites, which shows potential to fulfil those requirements.

## Graphical abstract

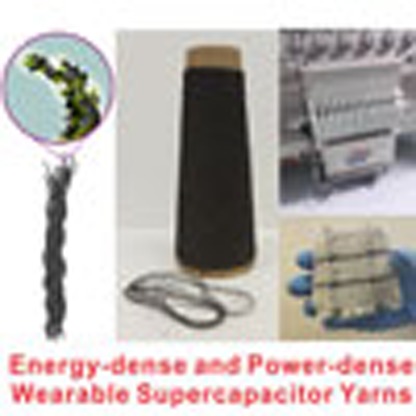

In recent years, a rapidly increasing research progress has been made in wearable electronics because of its remarkable application potentials in energy harvesting, micro-robotics, electronic textiles, epidermal and implantable medical devices^1^,^2^,^3^,^4^. One critical challenge identified in this field is how to develop lightweight, wearable and high-performance energy-storage devices, which are indispensable parts to power the functioning devices in a wearable system. Among the many energy-storage devices, flexible supercapacitors (SCs) are promising candidates because of their quick charge–discharge capability, long life cycle and good safety^5^,^6^,^7^,^8^,^9^,^10^,^11^,^12^,^13^. In particular, when compared with two-dimensionally shaped thin-film SCs or fabric SCs, one-dimensionally shaped SC yarns (in some literature, they are also referred as SC fibres or SC wires) can maximize the mechanical flexibility and capacitance density, because of their small volumes, and can be easily assembled into various structures for design innovation^10^,^14^,^15^,^16^,^17^,^18^,^19^,^20^,^21^,^22^,^23^.

In this field, pure metal wires have been demonstrated as effective current collectors for making high capacitance and high power-density SC yarns because of their high conductivity. Early examples include polyaniline-coated stainless steel wires^24^, reduced graphene oxide (RGO)-coated Au wires^25^ and pen ink-decorated metal wires^26^. Very recently, Lee *et al*.^27^ reported the fabrication of two-ply SC yarns using Pt microfibres and poly(3,4-ethylenedioxythiophene)/carbon nanotube (CNT) composite fibres. The volumetric capacitance and power density were as high as 179 F cm^−3^ and 40 W cm^−3^, respectively. However, the energy density of SCs using pure metal wires was still low. These metal wires, for example, Pt, also make the device very heavy and costly.

To improve the lightweight and energy density, carbon-based SC yarns comprising carbon fibres, CNTs, RGOs, redox-active metal oxides or their composites have been extensively studied^17^,^28^,^29^,^30^,^31^,^32^. Although the energy density could be significantly improved to ∼6 mWh cm^−3^, the power density dropped markedly because of the relatively low conductivity of these carbon materials. In recent times, this problem was partially overcome by Yu *et al*.^33^. They reported an SC yarn, which could simultaneously achieve high power density (1,085 mW cm^−3^) and energy density (6.3 mWh cm^−3^) by using core-shell composite electrodes made of highly conductive single-walled CNT (SWCNT) core and porous RGO shell. Nevertheless, SWCNT is still considered a very expensive material and the core-shell fabrication requires a complicated process, which hamper the scale-up application of this device.

To this end, it is still a significant challenge on how to fabricate lightweight and durable SC yarns possessing a combination of high capacitance, high power density and high energy density with cost-effective materials and a scalable fabrication process^34^. To address this challenge, we report herein for the first time the development of low-cost ‘graphene/metallic textile' composite electrodes that lead to high-performance all-solid-state SC yarns. In the materials design, metallic textile yarns made of bundled Ni-coated cotton fibres are used as flexible, highly conductive, lightweight and durable current collectors of the electrodes, while RGO nanosheets coated on the inner and outer spaces of the metallic textile yarns provide remarkable electrochemical double-layer capacitance of the electrodes.

Importantly, this hierarchical composite electrode structure not only creates large surface areas for RGO uptake and exposure to the electrolyte but also allows effective charge transportation between graphene and the Ni surfaces. Therefore, all solid-state SC yarns made of these RGO/Ni cotton composite electrodes can simultaneously achieve high energy density (similar to those of commercial thin-film lithium batteries) and high power density with good life cycle and bending fatigue retention. More importantly, the composite electrodes are fabricated with solution processes that are fully compatible with high-throughput textile processing. These SC electrodes and yarns can be readily assembled with textile embroidery and weaving technologies for wearable applications. To the best our knowledge, this is the first report on using low-cost metal-coated textile for making high-performance SCs.

## Results

### Ni-coated cotton yarns as current collectors

**Figure 1:**
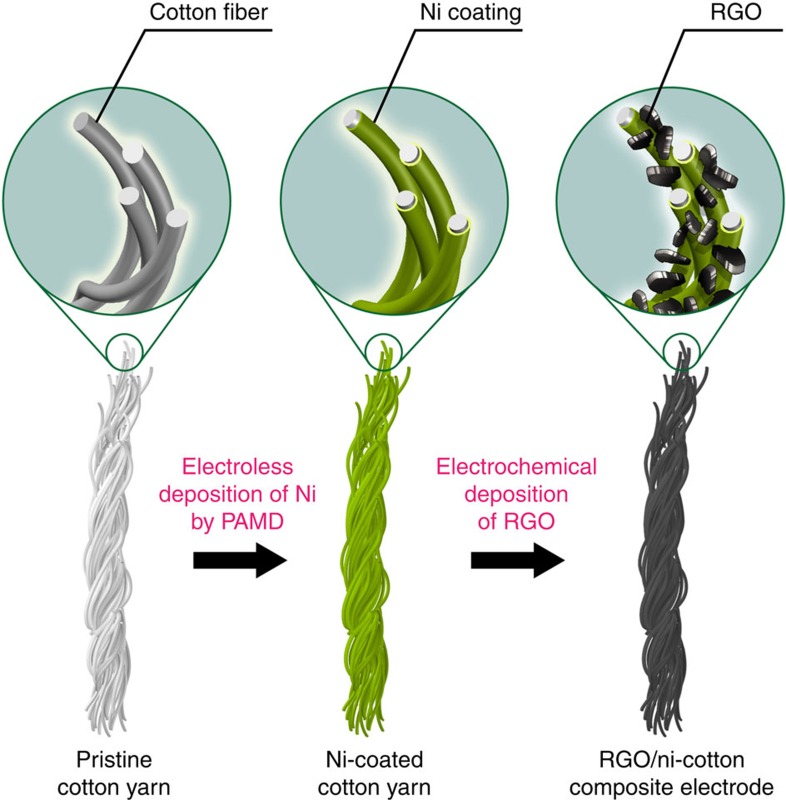
Schematic illustration. Schematic illustration of the fabrication of RGO/Ni cotton yarn composite electrodes.

Conductive textiles can be fabricated by depositing a thin layer of metal on the textile surface via galvanic deposition, atomic layer deposition, electrochemical deposition, electroless deposition (ELD) and others^35^,^36^,^37^,^38^,^39^. Among these techniques, ELD is particularly attractive, because it does not require expensive equipment and can be carried out under ambient conditions at a large scale^40^,^41^,^42^. In this work, Ni-coated cotton yarns were fabricated by a ‘polymer-assisted metal deposition' method (Fig. 1)^43^. In a typical experiment, commercially available, pre-cleaned cotton yarns were dipped into an ethanoic solution of poly[2-(methacryloyloxy)ethyl trimethylammonium chloride-*co*-3-(trimethoxysilyl)propyl methacrylate] [P(METAC-*co*-MPTS)]. After hydrolysis and curing steps, ∼10-nm-thick P(METAC-*co*-MPTS) was covalently grafted onto the cotton surfaces^41^. Subsequently, the copolymer-grafted cotton yarns were immersed into an aqueous solution of (NH_4_)_2_PdCl_4_, where PdCl_4_^2−^ were loaded onto the copolymer layer through the strong ionic interactions with the quaternary ammonium groups. Finally, the samples were immersed into an ELD bath of Ni for a certain time, in which a thin layer of Ni was deposited on the surface of the cotton yarn.

**Figure 2:**
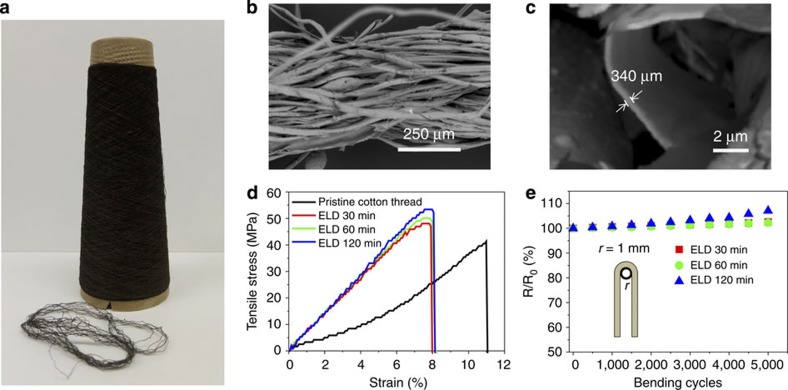
Ni-coated cotton yarns as current collectors. (**a**) Digital image of a 500-m-long Ni-coated cotton yarn wound on a spinning cone. (**b**) Low-magnification and (**c**) high-magnification cross-sectional scanning electron microscopic image of a Ni-coated cotton yarn made at ELD time of 60 min. (**d**) Stress–strain curves and (**e**) electrical resistance (bending radius=1 mm) of Ni-coated cotton yarns.

This fabrication was highly scalable, because the process was performed in a solution manner. Figure 2a shows an as-made 500-m-long Ni-coated cotton yarn that was wound on a spinning cone. The thickness of the Ni-coated cotton yarns was 0.45 mm in average. As chemicals were able to penetrate into the inner space of the cotton yarns in this wet processing, Ni was uniformly and densely coated on the surfaces of both the outer and inner cotton fibres of the yarn (Fig. 2b and Supplementary Fig. 2). The thickness of the Ni coating increased from ∼260 to ∼650 nm as the ELD time increased from 30 to 120 min (Supplementary Fig. 1 and Supplementary Table 1). These Ni cotton yarns are much lighter than pure metal yarns used in some literature reports^24^,^25^,^26^,^27^. For example, the density of 60-min ELD Ni-coated cotton yarn was 2.33 g cm^−3^, which is only 25% of that of pure Ni yarns (8.91 g cm^−3^) and 10% of that of pure Pt yarns (21.45 g cm^−3^). Stress–strain tests showed that the Ni-coated cotton yarns were ∼190% stronger than pristine cotton yarns (Fig. 2d).

Importantly, the Ni-coated cotton yarns processed high conductivity, whereaile keeping the textile-like flexibility. The initial electrical resistances of the Ni-coated cotton yarns were ∼2.2, 1.6 and 1.3 Ω cm^−1^ when the ELD times were 30, 60 and 120 min, respectively. These yarns were bent and unbent 5,000 cycles to evaluate the fatigue resistance under wearable conditions. At large bending radius (*r*), we did not observe obvious change in the electrical resistance for all the Ni-coated cotton yarns. Figure 2e shows the resistance change in bending tests with the smallest bending radium, *r*=1 mm. After 5,000 bending cycles, the electrical resistance increased by 7.1% for samples of 120-min ELD and only 2% for samples of 30- and 60-min ELD. The slightly poor performance for samples of 120 min deposition time may be attributed to the small cracking in the thick Ni layer during the bending fatigue test (Supplementary Fig. 3). Considering the overall performance in weight, electrical resistance and flexibility, we used Ni-coated cotton yarns made with 60-min ELD as the current collectors of the composite electrodes, which are discussed in detail below.

### RGO/Ni cotton composite electrodes

**Figure 3:**
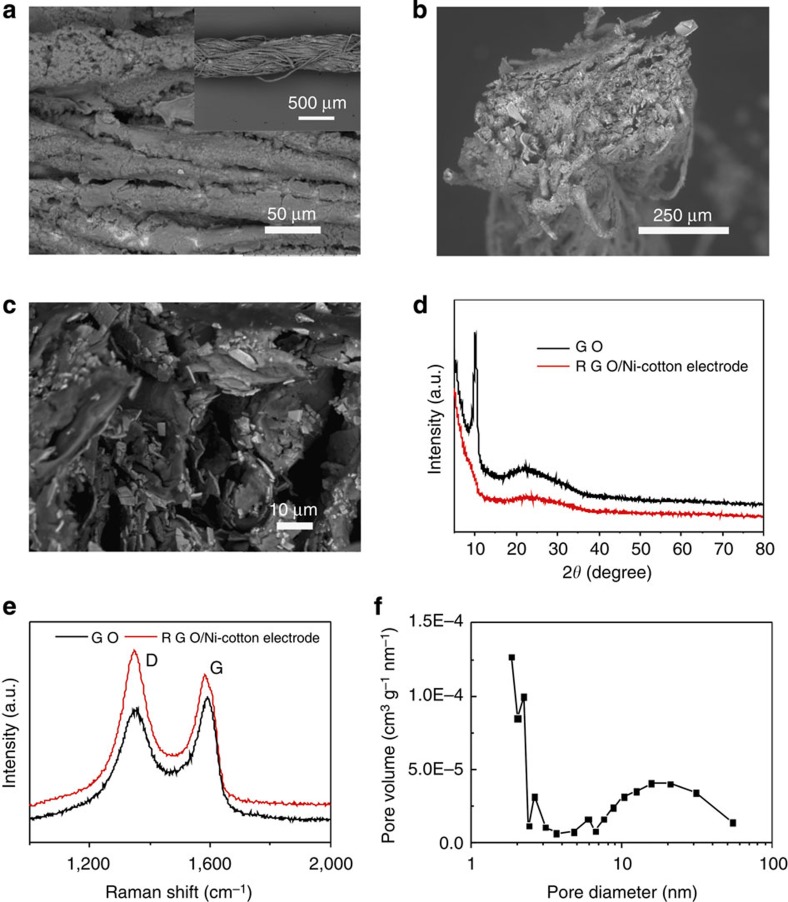
Characteristics of the RGO/Ni cotton composite electrode yarn. (**a**,**b**,**c**) Scanning electron microscopic images at different magnifications of a typical RGO/Ni cotton composite electrode made with 10-min RGO electrochemical deposition. The images show that RGO sheets are immobilized at the inner and outer spaces of the yarn. (**d**) X-ray diffraction, (**e**) Raman and (**f**) bulk heterojunction (BJH) pore distribution spectra of the RGO/Ni cotton composite electrode.

To fabricate the composite electrodes, graphene was deposited on the surface of Ni cotton yarns by electrochemically electrolysing 3 mg ml^−1^ graphene oxide (GO) aqueous suspension at an applied potential of −1.2 V for a certain time, in which the Ni cotton yarns were used as working electrodes (see Methods for details)^16^,^44^. A representative current–time curve of the electrodeposition was demonstrated in Supplementary Fig. 4. A hydrazine vapour reduction was applied after the electrochemical step. During the electrolysation and hydrazine vapour reduction, GO sheets were adhered onto the Ni surfaces and were reduced into conductive RGO. Energy-dispersive X-ray spectroscopy measurement showed that the coating was uniform all over the composite yarns (Supplementary Fig. 5). X-ray diffraction spectra showed that the representative peak of GO centred at 10.9° disappeared and a broad peak centred at 24.4° appeared after the reduction process, indicating that single- and few-layered RGO sheets were coated on the Ni cotton yarns (Fig. 3d). Raman spectroscopy study also proved the reduction process by monitoring the two signature bands around 1,349 and 1,583 cm^−1^, which were assigned to the D- and G-bands of carbon, respectively (Fig. 3e)^45^. The G-band is related to graphitic carbon and the D-band is associated with the structural defects or partially disordered structures of graphitic domains. The *I*_D_/*I*_G_ value was calculated to be 0.93 for GO and 1.15 for RGO, indicating that the GO sheets were reduced and their conjugated structures were partly restored^45^.

It was observed that RGO flakes penetrated into the multiple interval space among the individual fibres of the Ni cotton yarns. With an increasing electrochemical deposition time, the RGO coating became thicker and denser (Fig. 3 and Supplementary Fig. S6). We found that the Ni cotton yarn was fully covered by RGO flakes after 20-min electrochemical deposition. Brunauer–Emmett–Teller measurements were carried out to evaluate the porous structures of the RGO coatings at different deposition time (Fig. 3f and Supplementary Table 3). It is worth noting that these values were calculated based on the mass of the entire composite electrode, which included the cotton yarn, the Ni coating and the immobilized RGO sheets. The surface area of the composite electrodes also increased as a function of electrodeposition time. However, it should be noted that the trend is not linear. The surface area first increased rapidly from 2.77 to 7.11 m^2^ g^−1^ after 10-min electrodeposition. Next, the increment slowed down seriously: only another ∼7% increase in surface area at 20-min electrodeposition. On the other hand, it was observed that the pore volume of the composite electrode reached a maximum at 10-min electrodeposition time (0.015 cm^3^ g^−1^) and longer electrodeposition (20 min) showed a negative impact on the pore volume.

**Figure 4:**
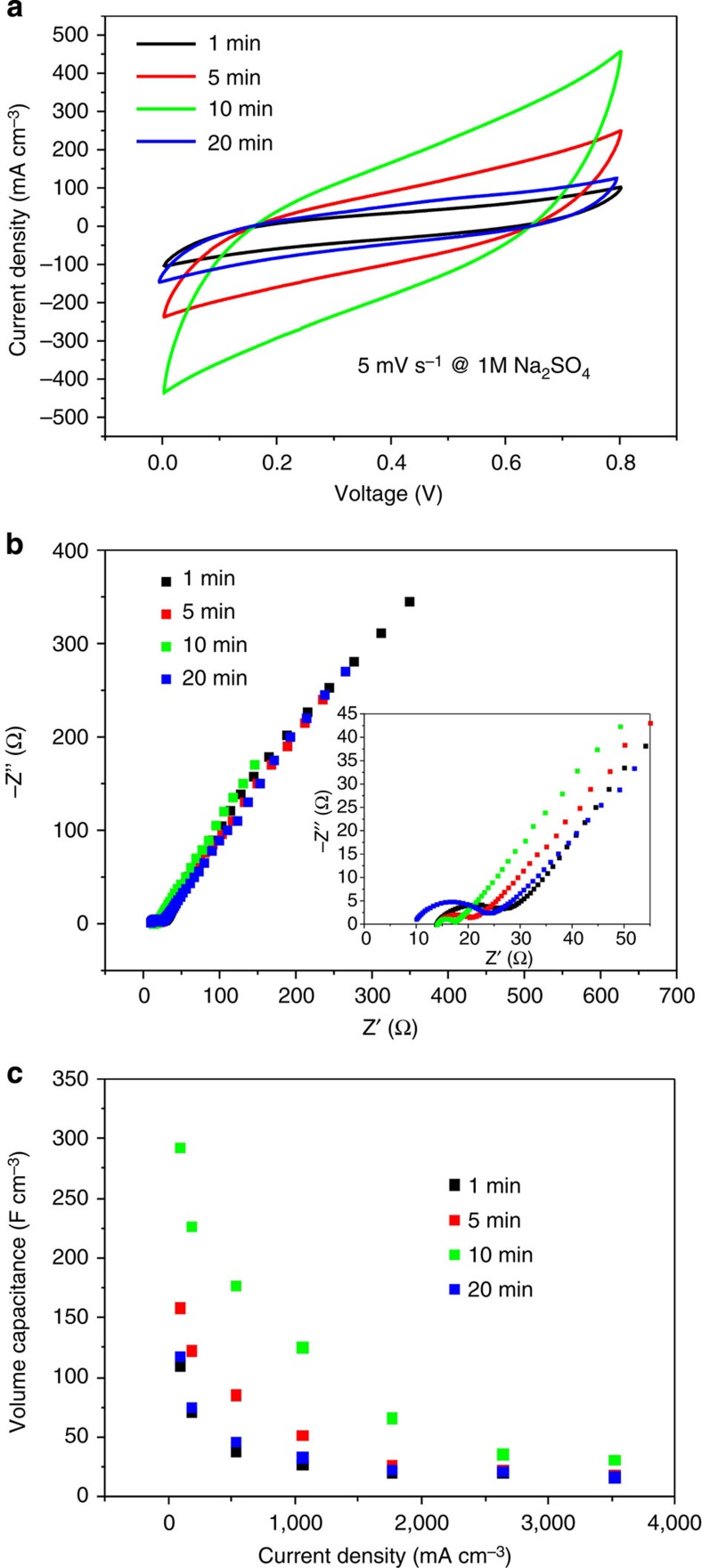
Electrochemical behaviours of RGO/Ni cotton composite electrodes in 1 M Na_2_SO_4_. (**a**) CV curves, (**b**) electrochemical impedance plots and (**c**) volumetric-specific capacitances of RGO/Ni cotton composite electrodes made at different RGO deposition time from 1 to 20 min.

To evaluate the electrochemical properties of the composite electrode, we assembled one pair of RGO/Ni cotton yarns in parallel in 1 M Na_2_SO_4_ solution. This two-electrode measurement is believed to better evaluate the true performance of electrode compared with a three-electrode measuring system. Regardless of the electrochemical deposition time of RGO of the composite electrodes, cyclic voltammetry (CV) curves of all the as-made assemblies exhibited slightly slopped rectangular-like shapes (Fig. 4a). Electrochemical impedance spectroscopies (EIS) of the devices showed small semicircles at high-frequency regions, which was due to small charge-transfer resistance, and linear behaviour in the low-frequency regions, also revealing an ideal capacitive behaviour of the electrode (Fig. 4b). The RGO/Ni cotton electrode with 10-min RGO deposition not only had a relatively low equivalent series resistance but also showed much shorter Warburg region portion compared with others (1-, 5- and 20-min samples), which indicated better ion diffusion efficiency due to smaller contact resistance among the material and ion^46^. This was because the amount of RGO in the composite electrode reached a high level and the porosity and the space in the composite electrodes still remained at the same level, as discussed above. When the deposition time was <10 min (1 and 5 min), the amount of RGO was less, leading to smaller capacitance and higher impedance. When the deposition time was too long (20 min), the RGO layer was too dense so that the diffusion of electrolyte was prohibited, also leading to smaller capacitance and higher impedance. The resistance, which was calculated from the intercept of the plot in the EIS spectrum, increased from 17 Ω for deposition time of 10 min to 25 Ω when using composite electrodes made with 20-min electrochemical deposition of graphene. Therefore, considering the above evidences, the optimized electrochemical deposition time of RGO was determined to be 10 min.

The specific capacitance of composite electrodes was then calculated based on the electrochemical measurements discussed above. Recently, it was shown that volumetric capacitance gave a more accurate picture of the performance of an SC compared with gravimetric values^11^,^47^. This is even more relevant in the case of yarn-typed SCs, because the mass of the active materials absorbed on the electrode is negligible compared with the weight of the entire device. In the calculation, we considered our composite electrode as a cylinder, with a cross-sectional diameter being the thickness of the composite electrode. Same as the previous analysis, devices made with 10-min electrochemical deposition of RGO showed the highest volumetric capacitances (Fig. 4c). The specific volumetric capacitance of the composite electrode (*C*_v,electrode_) was calculated using their galvanostatic discharge curves as follows:


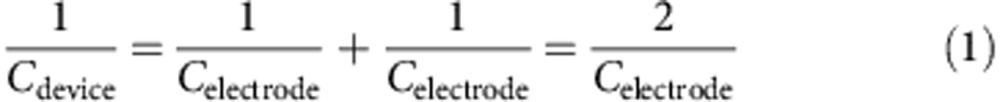



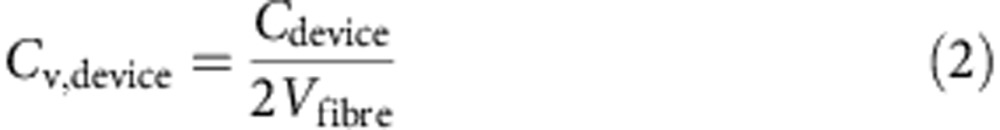



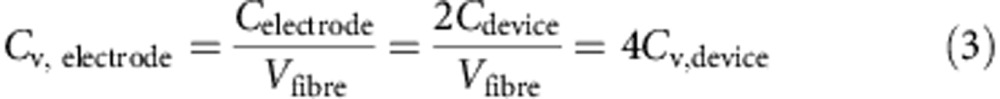


where *C*_v,device_ is the specific volumetric capacitance of the device in the two-electrode configuration and *V*_fibre_ is the volume of the single fibre.

It was observed that *C*_v, electrode_ dropped with increasing current density, a typical characteristic of SCs. This capacitance decay in high current densities was most likely to be due to the insufficient ionic transport in the charge/discharge process. The highest *C*_v,electrode_ of liquid–SC yarn was 292.3 F cm^−3^ at a current density of 87.9 mA cm^−3^, which is among the best values reported in the literature^33^. The degree of capacitance decay was also similar. For example, Yu *et al*.^33^ reported that the specific volumetric capacitance decayed to 56% of its initial capacitance at 800 mA cm^−3^ in H_2_SO_4_ electrolyte; our specific volumetric capacitance decayed to 44% of its initial capacitance at 1,000 mA cm^−3^ in Na_2_SO_4_ electrolyte.

### Solid-state SC yarns with RGO/Ni cotton composite electrodes

**Figure 5:**
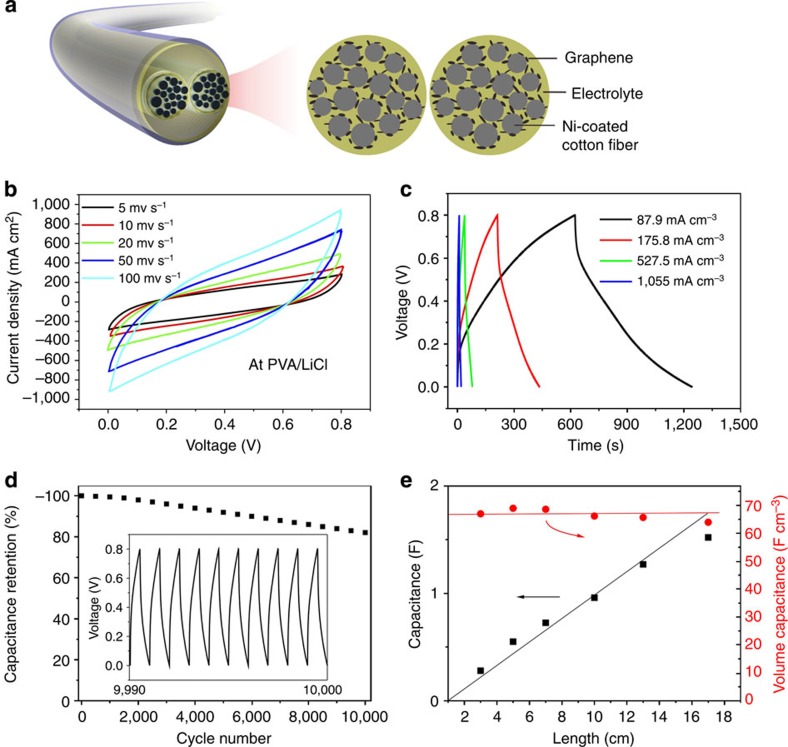
Performances of solid-state SC yarns. (**a**) Schematic illustration of the structure of one SC yarn. (**b**) CV curves of the device at scan rates ranging from 5 to 100 mv s^−1^. (**c**) GCD curves of the device at different current densities. (**d**) Cycle life of the device. The inset is the GCD curve from the 9,990th to 10,000th cycle. (**e**) Device capacitance as a function of the device length.

Based on the remarkable capacitive behaviour of the optimal RGO/Ni cotton composite electrodes (10-min electrochemical deposition of RGO), we fabricated a wearable solid-state SC yarn with a polyvinyl alcohol (PVA)/LiCl gel, which acted as both the electrolyte and an effective separator of the device (Fig. 5a). The total volume of one 3.5-cm-long SC yarn, including two composite electrode yarns and the surrounding solid electrolytes, was estimated to be 0.011 cm^−3^. As expected from the electrode characteristics, the CV curves of the SC yarn at scanning rates ranging from 5 to 100 mV s^−1^ also showed slightly slopped rectangular-like shapes within a potential window from 0 to +0.8 V (Fig. 5b). The shape at high scan rates were similar to those at low scan rates without obvious distortion, which indicates a high rate performance and efficient ionic and electronic transports within the electrode materials^48^,^49^. The galvanostatic charge/discharge (GCD) curves at different current densities (Fig. 5c) exhibited triangular shapes with a columbic efficiency of ∼99%, indicating excellent reversibility of the device and good charge propagation between the two composite electrodes. The SC yarn demonstrated good life cycle stability: 82% of its initial capacitance was retained after 10,000 cycles of GCD measurements at a current density of 439.6 mA cm^−3^ (Fig. 5d).

In particular, the capacitance of the SC yarn scaled up proportionally in a first order as its length increases. As shown in Fig. 5f, varying the length of the SC yarn from 3 to 17 cm resulted in a linear increase in the device capacitance from 0.28 to 1.52 F. Accordingly, the derived linear capacitance of the SC yarn is ∼0.11 F cm^−1^, which is almost 20-fold higher than the ever-highest values of coaxial CNT/carbon fibre SC (∼6.3 mF cm^−1^)^50^ and RGO/CNT fibre SC (∼5.3 mF cm^−1^)^30^.

**Figure 6:**
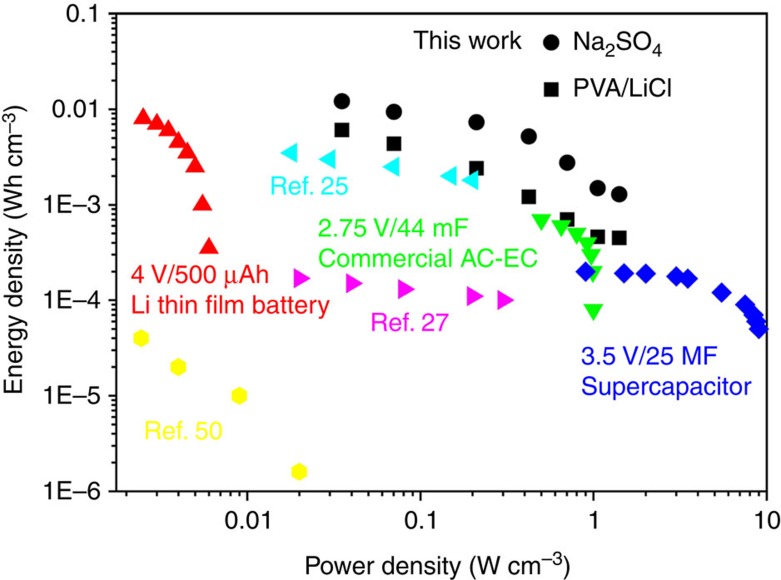
Ragone graph of different SC yarns and some commercial devices. Commercially available 4 V/500 μAh Li thin-film battery^50^, a commercially available AC-EC^12^, a 3.5 V/25 mF SC^50^ and some published experimental data are listed in the graph.

More importantly, the volumetric energy density (*E*_v_) and power density (*P*_v_) of the SC yarn, which are more meaningful parameters for evaluating the energy-storage performance of the device, could both reach remarkably high levels. For ease of comparison, the *E*_v_ and *P*_v_ of as-made SC yarns, as well as some representative devices from the commercial markets and latest literatures listed in Supplementary Table 2, are plotted into a Ragone graph as shown in Fig. 6. Evidently, the *E*_v_ and *P*_v_ of the as-made SC yarn position at the upper right corner of the Ragone plot. The maximum *E*_v_ of the SC yarn is 6.1 mWh cm^−3^, which is approximately eightfold higher than that of commercially available 2.75 V/44 mF SC^12^ and 30-fold higher than that of 3.5 V/25 mF SC^51^, and is even comparable to that of 4 V/500 μAh thin-film lithium battery (0.3–10 mWh cm^−3^). This energy density is among the highest values of recently reported yarn-based SCs (Fig. 6b and Supplementary Table 2). The maximum *P*_v_ of our SC yarn is 1,400 mW cm^−3^, which is also among the best value compared with the other published data (Supplementary Table 2).

### Wearable applications of SC yarns

**Figure 7:**
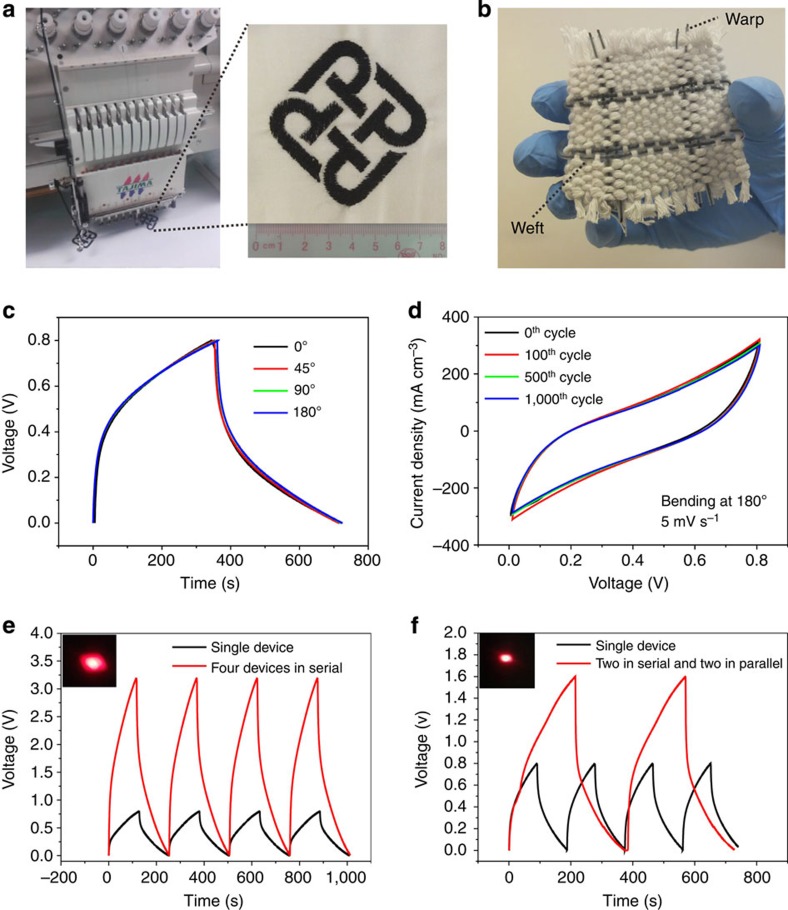
Wearable applications of solid-state SC yarns. (**a**) Digital images of embroidery logos of The Hong Kong Polytechnic University using the composite electrode yarns. (**b**) A woven fabric made with solid-state SC yarns. (**c**) GCD curves of solid-state SC yarns at different bending angles. (**d**) CV curves of the device at different bending (180° bending angle) cycles. Galvanostatic charge/discharge curves of four SCs connected (**e**) in series and (**f**) in a combination of series and parallel. Insets in **e** and **f** are digital images of a light-emitting diode lightened by the respective tandem SCs.

As the SC yarn is assembled on the basis of textile materials and solid-state electrolyte, it is ideal for different wearable applications. For example, the composite electrodes could be directly used as threads in commercial embroidery machines. As proof-of-concept, we embroidered the logo of The Hong Kong Polytechnic University on a piece of cotton fabric (Fig. 7a and Supplementary Movie 1). It should be noted that raw cotton yarns were not suitable for embroidery, because they were not strong enough to overcome the high tensile strength applied during the process. The RGO/Ni cotton composite electrode yarns, however, possessed higher tensile strength (Fig. 2) due to the metallic coating, which makes it suitable for embroidery.

In a second example, we used our solid-state SC yarns as weft and warp yarns, which were woven into a fabric with other pristine cotton yarns in a conventional weaving process (Fig. 7b). To test the stability of as-made SC yarns for flexible and wearable applications, we bent the device at different angles ranging from 0° (unbent) to 180° (folded) while measuring the GCD property. As shown in Fig. 7, bending of the device had little effect on its capacitive behaviour. The device could be bent arbitrarily without degrading its performance. This device was then folded and unfolded for 4,000 cycles to simulate a wearing condition. The scanning electron microscopic observation showed no obvious change in the surface morphology of the RGO/Ni cotton composite electrode after the bending tests (Supplementary Fig. 7). Only 5% decay in volumetric capacitance was observed, while the columbic efficiency was kept in the range of 98%–100% throughout the entire test (Supplementary Fig. 8). Such an excellent durability of the device can be attributed to the high mechanical flexibility of the RGO/Ni cotton composite electrodes along with the interpenetrated gel electrolyte. The electrolyte solidifies during the device assembly and acts similar to a glue that holds all the device components together, which also improves the mechanical integrity and increase its life cycle even when tested under extreme bending conditions.

In a third example, the SC yarns were connected either in series, in parallel or in a combination of both, to demonstrate the flexibility to meet operational voltage and/or power requirements of different wearable devices. Compared with the single SC yarn that operated at 0.8 V, four SC yarns connected in series exhibited a 3.2 V charge/discharge voltage window (Fig. 7e). In the parallel assembly, the output current increased by a factor of 4 and the discharge time was fourfold that of a single device when operated at the same current density (Supplementary Fig. 9). As expected, when the four SC yarns were combined two in series and two in parallel, both the output voltage and discharge time doubled under the same charge/discharge current (Fig. 7f). It is worth noting that this exceptional performance was achieved without using a voltage balance, which is often needed in series connection to prevent any device from going into an overbiased situation. These tandem SCs could be readily used for lighting light-emitting diodes that operated at a minimum voltage of 1.7 V. For instance, three 3.5-cm-long SCs connected in series were able to power a red light-emitting diodefor >40 s (Supplementary Movie 2).

## Discussion

In conclusion, the wearable solid-state SC yarns made of RGO/Ni cotton composite electrodes outperform other yarn-type SCs in the literature because of their combinatorial advantages of high capacitance, high energy density, high power density, excellent mechanical durability and flexibility, and remarkable scalability in the materials synthesis and device fabrication. As-made high-performance SC yarns have been used in embroidery and weaving technologies for easy and high-speed integration into many different wearable applications. The novel properties are ascribed to the hierarchical structures of RGO/Ni cotton composite electrodes in the following aspects.

First of all, Ni-coated cotton yarns retain the excellent flexibility and light weight of the pristine cotton yarns, while they excel in strength and electrical conductivity. In comparison, although pure metal fibres and wires have excellent conductivity and strength, their heavy weight and rigidity limit their application for wearable electronics. Therefore, Ni-coated cotton yarns are ideal scaffolds and current collectors for the wearable SCs.

Second, solution-processed metal coating and RGO deposition methods ensure the deep penetration of chemicals into the cotton yarn, resulting in a uniform coating of these electrode materials on the surfaces of individual cotton fibres at the inner and outer positions of the cotton yarn. This hierarchical structure can effectively prevent restacking of RGO sheets and collapsing of transporting channels. As a consequence, the composite electrode possesses not only large surface area of active materials but also sufficient access of the electrolyte to form electrochemical double layers. To prove the efficient utilization of the surface area of the RGO sheets, we measured the specific gravimetric capacitance of RGO in our wearable SC yarns. According to the Lambert–Beer law (*A*=*αCL*), where the absorption *A* of the solution equals to the multiplication of the absorption coefficient *α*, the cell length *L* and the concentration *C*, the concentration of the as-made RGO solution can be determined (*A*/l≈0.5 m^−1^ for sodium dodecyl benzene sulfonate (SDBS) at 0.5 mg ml^−1^)^52^. Herein, the absorption coefficient *α* was determined by measuring the absorption of different concentrations of known RGO solutions (with 0.5 mg ml^−1^ SDBS in water) at 660 nm and its value is determined to be 876 ml mg^−1^ m^−1^ (Supplementary Fig. 10). To get the quantity of RGO in the composite electrode, we then added the as-made RGO/Ni-coated cotton yarns into 1 M HCl, to dissolve the coated Ni layer, and measured the absorption of the resulted RGO solution. To keep a good dispersion of RGO sheets in the solution, SDBS was also added with sonication. As such, the mass of RGO of a 10-cm-long composite electrode is 1.5 mg and the specific gravimetric capacitance is calculated to be 311 F g^−1^.

Third, from a physics point of view, one composite electrode yarn is made of tens of individual RGO/Ni-coated cotton fibre electrodes that are connected in series and parallel. This unique hierarchical architecture can minimize the diffusion distance of electrolytes between the bulk and the RGO surfaces, as well as the charge transport resistance between RGO and Ni surfaces. This advantage leads to high power density as compared with pure carbon materials based SC yarns.

Fourth, Ni and RGO are very low-cost materials compared with Pt wires and SWCNTs, which are used in the best-performing SC yarns in the literature. As GO sheets are made chemically by highly scalable solution method with low-cost raw materials (graphite), their production can be scaled up to meet commercial viability in real applications. In case of surface area, the outside of SWCNTs have a theoretical surface area of 1,320 m^2^ g^−1^. As for graphene, its theoretical surface area is 2,675 m^2^ g^−1^, because it is composed of all surface atoms at both sides of the layer. These advantages made GO a very attractive material for SC applications. The solution deposition processes of Ni and RGO are also compatible with industrial textile processing. Therefore, the fabrication of RGO/Ni cotton composite electrodes is highly scalable from both materials and fabrication point of views.

In principle, the concept of using hierarchical structured composite electrode should be applicable to other textile scaffolds and active materials. Therefore, we envision that the performance of SC yarns still has a large room to improve in the future. These SC yarns made of hierarchical composite electrode are ideal energy-storage devices for next-generation flexible, portable, and wearable electronics.

## Methods

### Preparation of Ni-coated cotton yarns

P(METAC-co-MPTS) was synthesized according to a literature report^41^. For ELD, commercial cotton yarns were cleaned using water and ethanol, and dried at 80 °C for 15 min. The precleaned cotton yarns were dipped into the ethanol solution of P(METAC-co-MTPS) and dried at 80 °C for 15 min. After that, samples were immediately incubated in an ammonia (95% relative humidity) atmosphere for ∼3 h at room temperature and then baked at 80 °C for 2 h. The samples were immersed into an aqueous solution of (NH4)_2_PdCl_4_ (0.5 mM) and deionized water for 10 min, respectively. Finally, the PdCl_4_^2−^ loaded cotton yarns were immersed into the ELD bath for a certain time and the corresponding Ni-coated cotton yarns were obtained^42^.

### Preparation of RGO/Ni cotton yarn composite electrodes

GO was prepared from natural graphite powder by a modified Hummer's methods^53^. The preparation of graphene-deposited cotton yarns was carried out by electrolysing 3 mg ml^−1^ GO aqueous suspension containing 0.1 M LiClO_4_, where the Ni-coated cotton yarn was used as working electrode, and Pt wire and Ag/AgCl (3 M KCl) were used as counter and reference electrodes, respectively. A constant potential of −1.2 V was applied. After electrochemical deposition, the samples were further reduced by hydrazine vapour at 60 °C for 3 h.

### Preparation of PVA/LiCl solid electrolyte

PVA/LiCl gel was prepared by mixing LiCl (6.3 g) and PVA (3 g) in deionized water (30 ml) and heated at 85 °C for 1 h under vigorous stirring.

### Assembly of SCs

For liquid devices, a thin, non-conductive cotton thread was wrapped around one of the electrode as a spacer, to prevent electrical short between the composite electrodes. In addition, this whole assembly was inserted in a flexible silicone tube, which was filled with 1 M Na_2_SO_4_ solution, to ensure effective exposure of the electrodes to the electrolyte. For solid devices, two electrodes were soaked with the PVA/LiCl solution and then allowed to solidify at room temperature for 6 h. Finally, they were assembled together in the silicone tube and heated at 45 °C for 12 h, to remove excess water in the electrolyte. No separator was used in the all-solid device. Finally, the device was sealed with epoxy.

### Characterization

The morphology and thickness of Ni on the cotton yarns were investigated by scanning electron microscope (TM3000, Hitachi). Stress–strain curves were conducted using an Instron 5565A. The CV and EIS measurements were carried out on an electrochemical workstation of a Solartron 1,255 frequency response analyser coupled with Solartron 1,287 electrochemical interface. The impedance spectra were recorded by applying a sine wave with 5-mV amplitude over a frequency range of 100 kHz to 0.01 Hz. *In-situ* conductivity–strain measurements were carried out by a two-probe method using M 2400 Keithley Multimeter. Nitrogen adsorption–desorption isotherms of graphene-coated cotton yarns were measured at 77 K using a Quantachrome Autosorb-6b static volumetric instrument. The samples were first degassed under high vacuum (<0.01 mbar). The specific surface area was calculated by the Brunauer–Emmett–Teller method. The Raman spectroscope with a 514-nm laser was calibrated with Si peak located at 520 cm^−1^ as the internal reference. X-ray diffraction characterization was conducted on X-ray powder diffractomerter (RigakuSmartLab) with monochromatic Cu Kα radiation (*λ*=1.5418 Å).

### Calculation of the electrochemical capacitance for cotton yarn electrode and all-solid-SCs

The cotton yarn SC is a symmetric two-electrode SC. The specific volumetric capacitance (*C*_v,electrode_) of the electrode in a two-electrode cell was calculated according to *C*_v,electrode_=2*C*/*V*_fibre_, where *C* is the measured capacitance in the two-electrode configuration and *V*_fibre_ is the volume of the single fibre. For the solid-state SC yarns, the volumetric capacitance (*C*_v_) of the device was derived from the equation: *C*_v_=*I* × *t* × *U*^−1^ × *V*^−1^, where *I* stands for charge–discharge current, *t* is the discharge time, *U* represents the potential window and *V* is the volume of two fibre electrodes including the electrolyte, which is equal to the cross-sectional area multiplied by the length (*L*) of overlapped portion of yarn electrodes. The length capacitance *C*_L_ was derived from the equation: *C*_L_=*I* × *t* × *U*^−1^ × *L*^−1^. The volumetric energy density (*E*_v_) and power density (*P*_v_) of the SCs can be obtained from *E*_v_=*C*_v_ × *U*^2^ × 7,200^−1^, *P*_v_=*E*_v_ × 3,600 × *t*^−1^.

## Additional information

**How to cite this article:** Liu, L. *et al*. Wearable energy-dense and power-dense supercapacitor yarns enabled by scalable graphene–metallic textile composite electrodes. *Nat. Commun.* 6:7260 doi: 10.1038/ncomms8260 (2015).

## Supplementary Material

Supplementary Figures and Tables Supplementary Figures 1-10 and Supplementary Tables 1-3.

Supplementary Movie 1 Embroidery of composite electrode yarns into university logos of The Hong Kong Polytechnic University. The black yarns are composite electrodes yarns.

Supplementary Movie 2 Lighting of a red LED with tandem SC yarns
