# Supplementary material for: Wearable energy-dense and power-dense supercapacitor yarns enabled by scalable graphene–metallic textile composite electrodes
Source: Nat Commun. 2015 Jun 11;6:7260. doi: 10.1038/ncomms8260 (PMC4490556; doi:10.1038/ncomms8260)
Supplement: Supplementary Figures and Tables — Supplementary Figures 1-10 and Supplementary Tables 1-3. [file ncomms8260-s1.pdf]

Supporting Information

Supplementary Figures

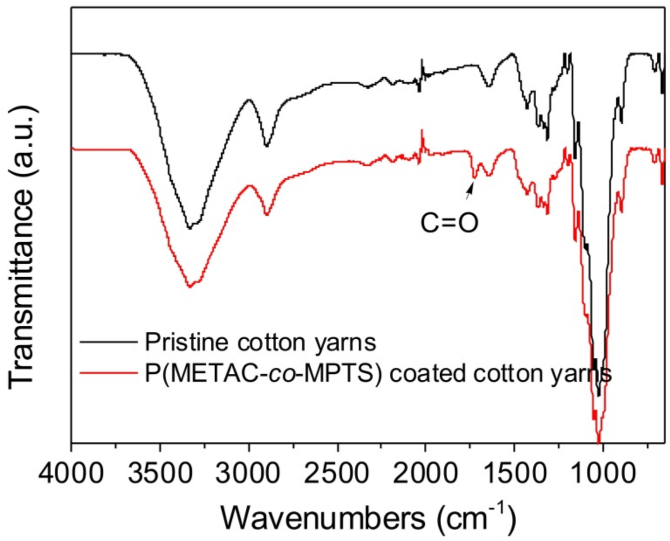

Supplementary Fig. 1 FT-IR spectra of pristine cotton yarns and copolymer coated cotton yarns. The appearance of C=O stretching (1737 cm^{-1}) indicates that the copolymer is coated on the surface of cotton yarns.

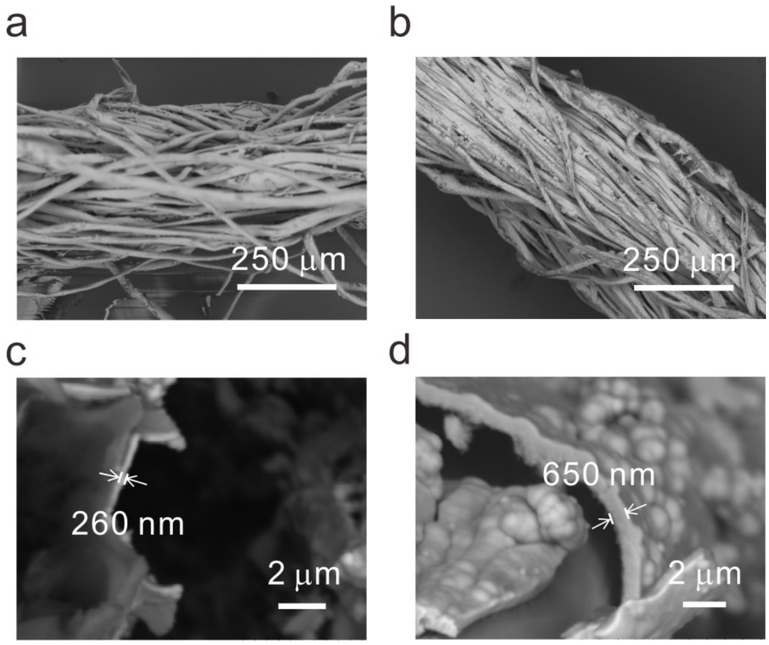

Supplementary Fig. 2 (a, c) SEM images of Ni-coated cotton yarns made with 30 min ELD. (b, d) SEM images of Ni-coated cotton yarns made with 120 min ELD.

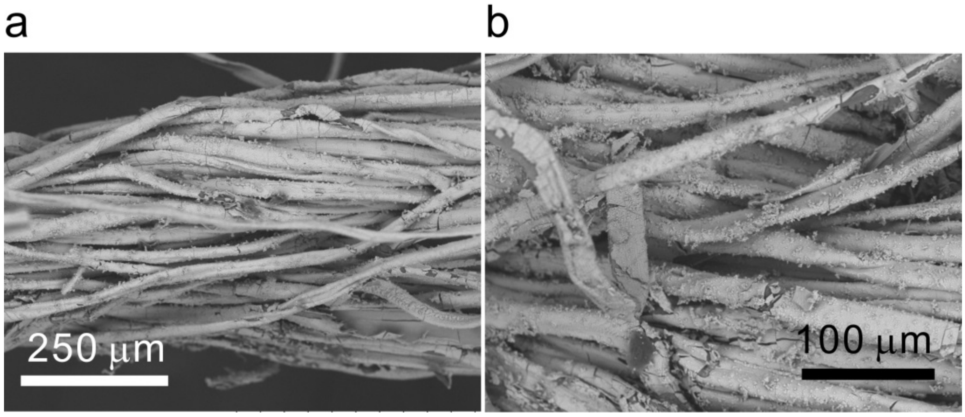

Supplementary Fig. 3 SEM images of Ni-coated cotton yarn after bending 5000 cycles at a bending radius of 1 mm.

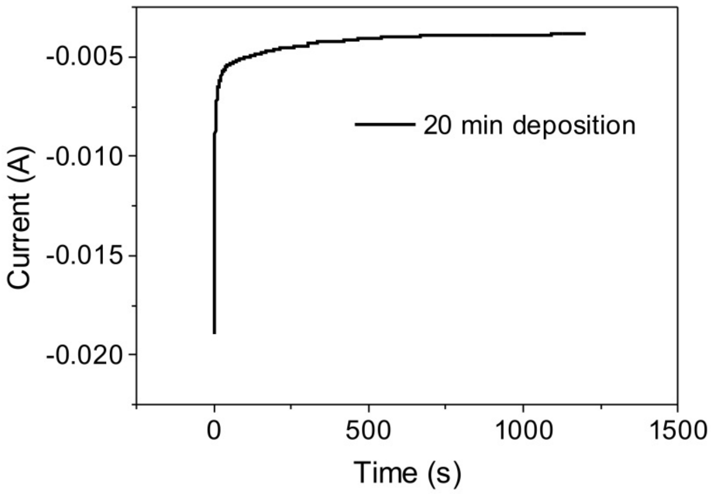

Supplementary Fig. 4 A representative *I*-*t* curve of the electrochemical deposition of RGO on Ni-coated cotton yarns at a constant potential of -1.2 V.

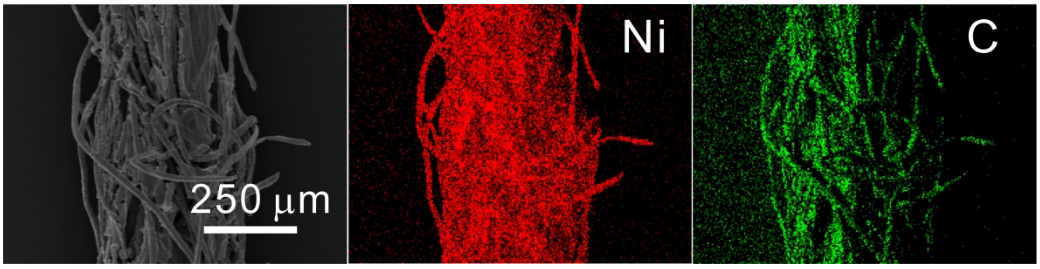

Supplementary Fig. 5 SEM images and EDX mapping of RGO/Ni-cotton composite electrode yarns made with 10 min electrochemical deposition.

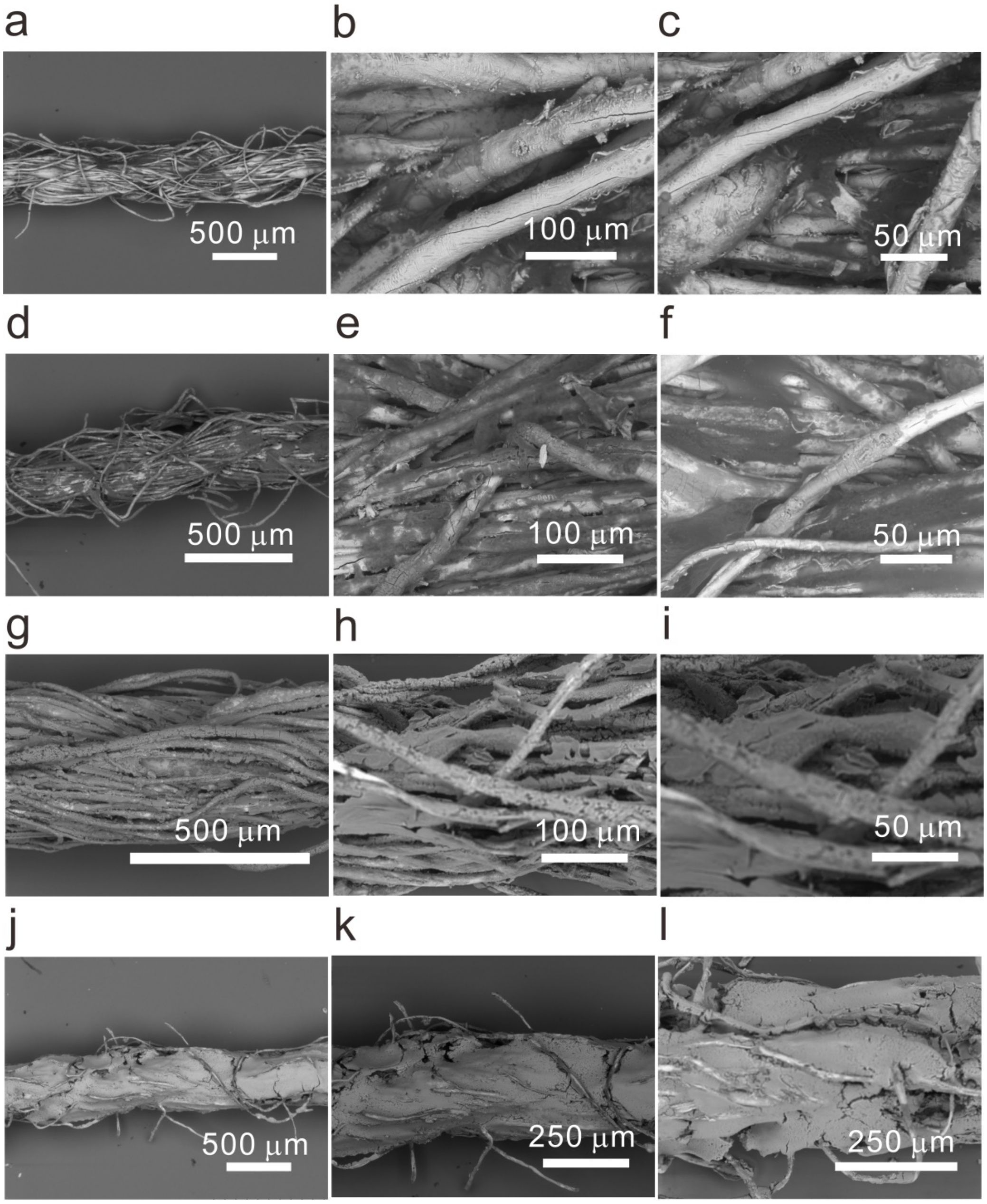

Supplementary Fig. 6 SEM images of RGO/Ni-cotton composite electrode yarns made with (a-c) 1 min, (d-f) 5 min, (g-i) 10 min and (j-l) 20 min electrochemical deposition.

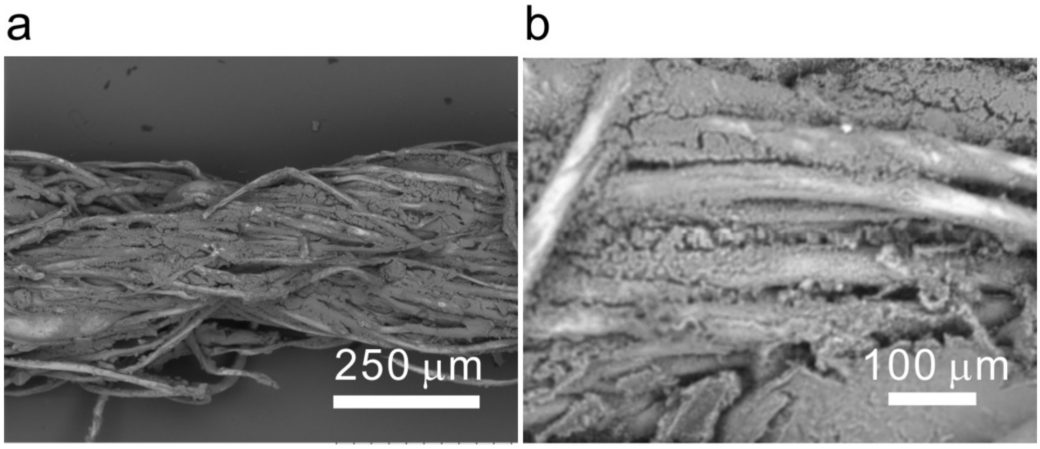

Supplementary Fig. 7 SEM images of RGO/Ni cotton yarn electrode after bending 4000 cycles at a bending radius of 1 mm.

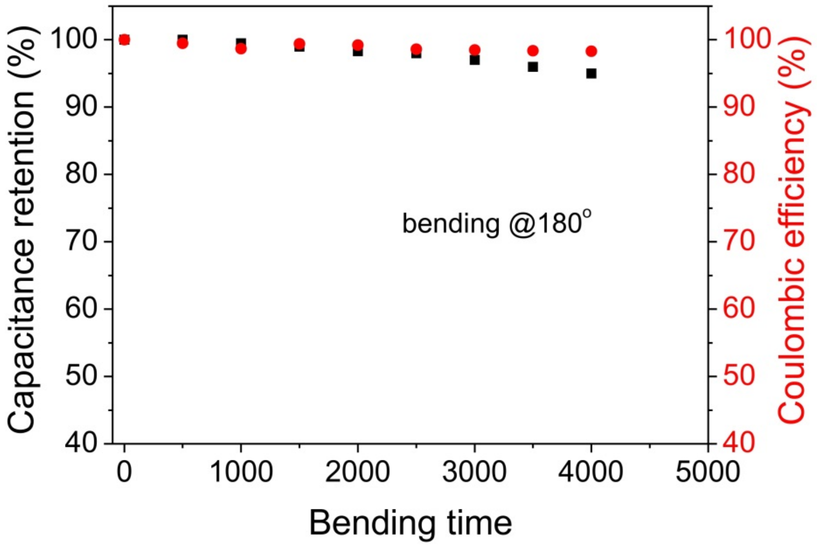

Supplementary Fig. 8 Capacitance retention (square) and coulombic efficiency (circle) of the device at different bending times.

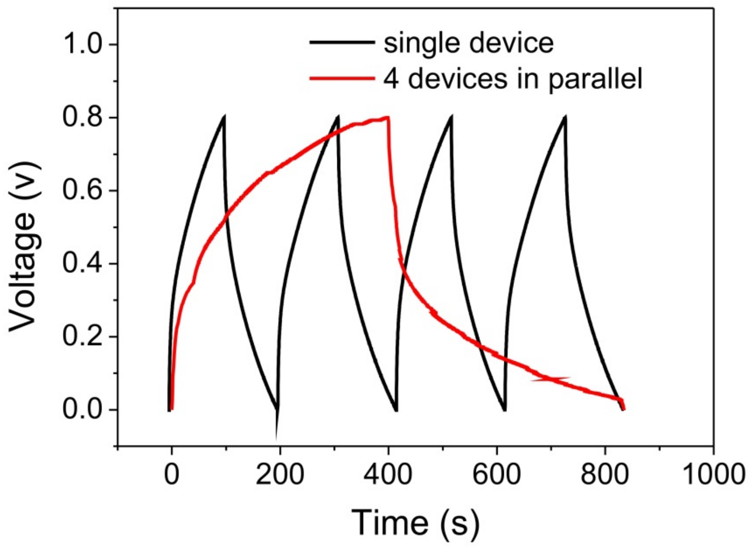

Supplementary Fig. 9 Galvanostatic charge/discharge curves for four supercapacitors connected in parallel.

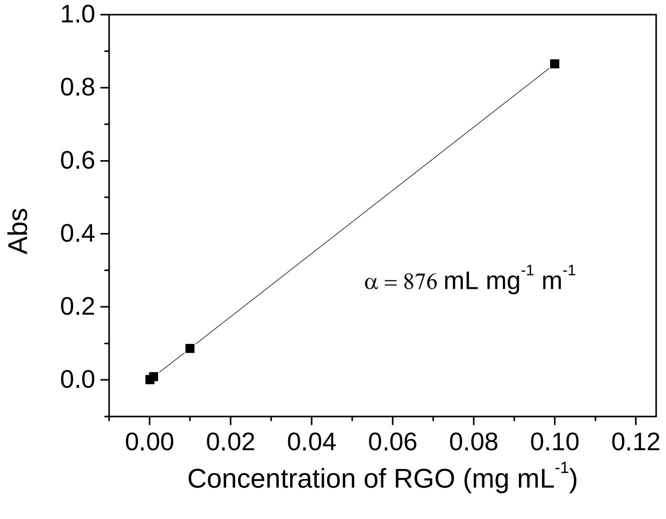

Supplementary Fig. 10 UV-vis measurement to determine the absorption coefficient of RGO aqueous solutions.

Supplementary Tables

Supplementary Table 1 Characteristic of the cotton yarn at different electroless deposition time.

ELD time (min)	Thickness (nm)	Resistance (Ω cm^{-1})	Density (g cm^{-3})
30	260±5	2.2±0.2	2.27±0.05
60	340±5	1.6±0.2	2.33±0.05
120	650±5	1.3±0.2	2.45±0.05

Supplementary Table 2. Comparison of electrochemical performance of yarn/fiber-based supercapacitors.

**Electrode materials**	**Electrolyte materials**	C_L **(mF cm^{-1})**	C_m **(F g^{-1})**	C_v **(F cm^{-3})**	**Cycle life**	**Bending fatigue**	P_v **(mW cm^{-3})**	E_v **(mWh cm^{-3})**	**Source**
RGO/Ni-cotton yarn	PVA/LiCl	110	311	68.2	92% @ 4000th ; 82% @ 10000th	95% @ 4000 cycles	1400	6.1	This work
CNT/Carbon fiber	PVA/H_3PO_4	6.3	-	-	94% @ 1000th	-	2.7	0.14	[44]
MnO_2/CNT fiber	$LiPF_6$	-	-	-	97% @ 1000th		790	1.7	[23]
CNT/MnO_2 yarn	PVA/KOH	-	-	25.4		100% @ 1000 cycles	127	3.52	[49]
MnO_2/ZnO/Carbon fiber	PVA/LiCl	-	-	0.325	87.5% @ 10000th	-	20	0.04	[50]
PPy/MnO_2/Carbon fiber	PVA/H_3PO_4	-	-	69.3	86.7% @ 1000th	-	400	6.16	[51]
RGO/SWCNT fiber	PVA/H_3PO_4	-	-	45	93% @ 10000th	97% @ 1000 cycles	1085	6.3	[28]
RGO/CNT	PVA/H_3PO_4	5.3	-	158	No decay after 2000th	98% @ 200 cycles	18	3.5	[25]
PEDOT/CNT/Pt wire	PVA/H_2SO_4	-	-	179	-	98% @ 2000 cycles	40000	1.4	[22]
CNT/PANI/Pt fiber	PVA/ H_3PO_4	0.24	86.2	-	-	-	-	-	[52]
Carbon/Cotton/Stainless Steel fiber	PVA/H_3PO_4/ SiWA	37	120	-	-	-	-	-	[53]

PPy, polypyrrole; SWCNT, single-walled carbon nanotube; PEDOT, poly(3,4-ethylenedioxythiophene); SiWA, silicotungstic acid.

Supplementary Table 3. Properties of RGO/Ni-cotton composite electrode yarns characterized by the nitrogen adsorption and desorption measurements.

	**Cotton yarns**	**Ni-coated cotton yarns**	**5 min RGO deposition**	**10 min RGO deposition**	**20 min RGO deposition**
**Surface area (m^2 g^{-1})**	2.77	3.37	5.60	7.11	7.66
**Pore volume (cm^3 g^{-1})**	0.01	0.008	0.015	0.015	0.014
**Pore size (nm)**	1.5~50	1.5~50	1.5~55	1.5~55	1.5~55
